# Genetics of auditory mechano-electrical transduction

**DOI:** 10.1007/s00424-014-1552-9

**Published:** 2014-06-25

**Authors:** Nicolas Michalski, Christine Petit

**Affiliations:** 1 Unité de Génétique et Physiologie de l’Audition, Institut Pasteur, Paris, France; 2 UMRS 1120, Institut National de la Santé et de la Recherche Médicale (INSERM), Paris, France; 3 Sorbonne Universités, UPMC Univ Paris 06, Paris, France; 4 Syndrome de Usher et autres Atteintes Rétino-Cochléaires, Institut de la vision, Paris, France; 5 Collège de France, Paris, France

**Keywords:** Cochlea, Hair bundle, Mechano-electrical transduction, Hair cell, Neurogenetics

## Abstract

The hair bundles of cochlear hair cells play a central role in the auditory mechano-electrical transduction (MET) process. The identification of MET components and of associated molecular complexes by biochemical approaches is impeded by the very small number of hair cells within the cochlea. In contrast, human and mouse genetics have proven to be particularly powerful. The study of inherited forms of deafness led to the discovery of several essential proteins of the MET machinery, which are currently used as entry points to decipher the associated molecular networks. Notably, MET relies not only on the MET machinery but also on several elements ensuring the proper sound-induced oscillation of the hair bundle or the ionic environment necessary to drive the MET current. Here, we review the most significant advances in the molecular bases of the MET process that emerged from the genetics of hearing.

## Introduction

**Fig. 1:**
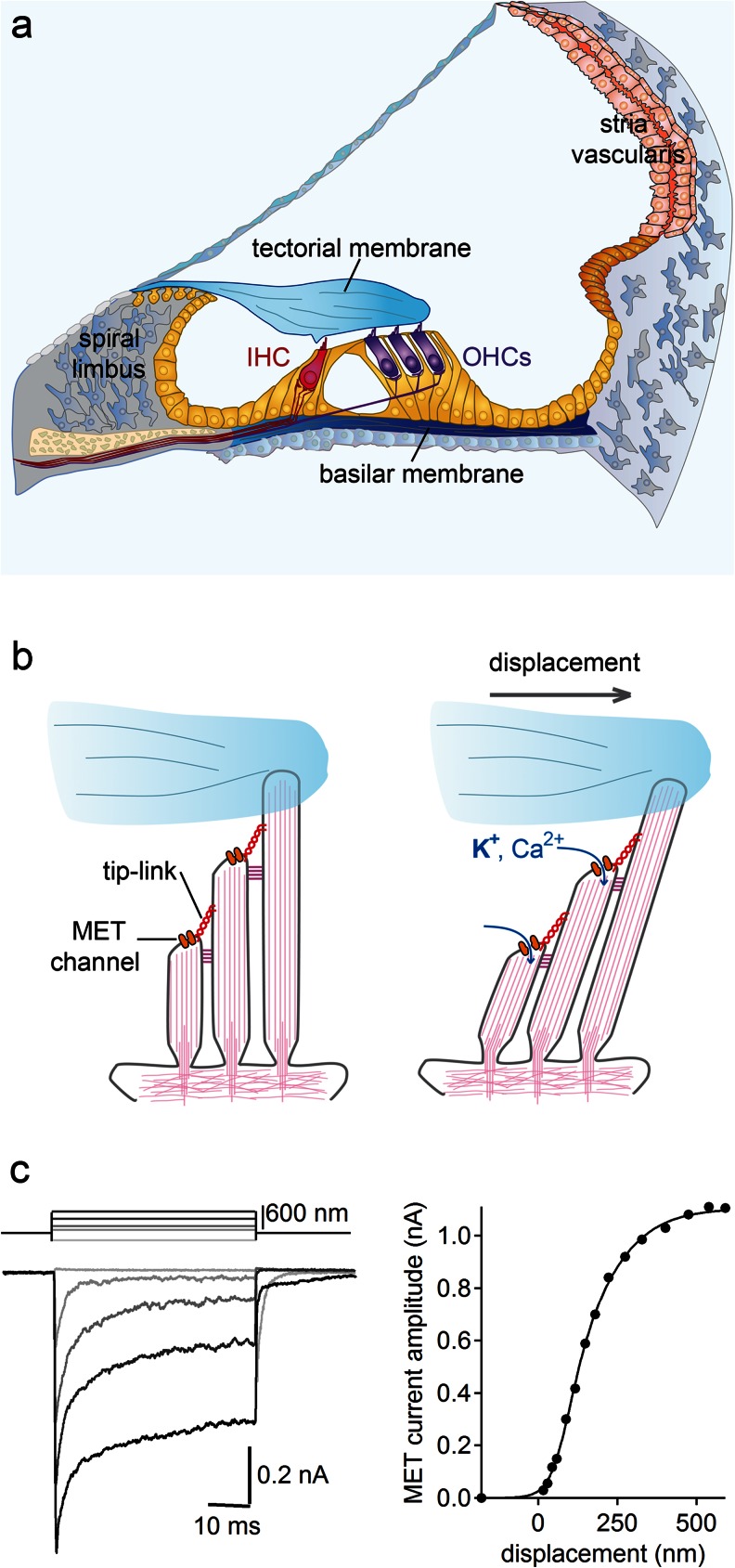
Auditory organ and MET. **a** Schematic cross-section of the cochlea. *IHC* inner hair cell, *OHC* outer hair cell. **b** Illustration of the stimulation of a mature OHC hair bundle. Stereocilia are maintained cohesive by top connectors (*purple*). The tallest row of stereocilia is anchored into the tectorial membrane. Upon hair bundle displacement towards this row (excitatory direction), high tension in the tip-links results in MET channel opening, leading to the entry of K^+^ and Ca^2+^ ions to the hair cell. **c** *Left* Example of transduction current recordings in an IHC, voltage clamped at −80 mV, of a P7 mouse while applying different displacement steps with a glass probe in the excitatory direction and a 180-nm step in the inhibitory direction (calibrated voltage command of the stimulator at the *top left*). *Right* Corresponding current–displacement plot fitted with a three-state Boltzmann relation

The ability of vertebrates to maintain their balance and sense sound vibrations is decisive for their survival. Although vertebrates live in various environments, they all make use of the same organelle, the hair bundle, that transduces mechanical information into an electrical signal in sensory hair cells. Hair cells are present in the neuromasts of lateral lines in fish and amphibian larvae, where they detect water movement; in the vestibular end organs, where they detect linear and angular acceleration; and in the auditory organs, where they detect sound pressure waves (Fig. 1a). Hair cells are also present in non-vertebrate organisms. For instance, the sea anemone, which belongs to the cnidarian phylum, uses hair cells located on its tentacles to detect zoo-plankton [228, 229]. The hair bundle is located at the apex of hair cells and is comprised of several rows of rigid, actin-filled microvilli, known as stereocilia, which are organised in a staircase pattern and maintained together by different types of links. One link, called the tip link, plays a major role in mechano-electrical transduction (MET). This oblique link connects the tip of each sterocilium to the lateral wall of the adjacent taller stereocilium. Upon mechanical stimulation of the hair bundle in the direction of the tallest stereocilia, i.e. the excitatory direction, tension in the tip links increases resulting in a higher probability of MET channel opening and cell depolarisation [87, 86, 164, 67, 10] (Fig. 1b, c). The biophysical features of these MET channels have been extensively studied. These cationic non-selective channels [23, 41, 154] have a large unitary conductance in the 100 pS range [43, 71, 154] and an extremely fast activation time constant [42, 205, 172], and are permeant to large organic cations such as choline and TEA [62, 154].

The first physical description of hair bundle functioning was reported in the late 1970s. However, the small number of hair cells in the inner ear (a few thousands) hampered molecular advances, as opposed to other sensory organs like the eye, which contains more than 100 million photoreceptor cells. In the early 1990s, human genetics, the efficiency of which is independent from the number of hair cells, emerged as the best approach to identify molecules involved in MET. Studies focused largely on the cochlea, the mammalian auditory organ, rather than on the vestibular organs because vestibular defects in humans are often compensated by the visual and proprioceptive systems. In addition, deafness is the most frequent sensory defect at birth (approximately one out of 700 newborns is affected by severe or profound hearing impairment). Currently, more than 120 deafness loci have been characterised, and around 80 genes responsible for isolated (non-syndromic) forms of sensorineural deafness have been identified (see the Hereditary Hearing Loss Homepage: http://hereditaryhearingloss.org). In addition, many more genes are involved in syndromic forms of sensorineural deafness. Pathophysiological studies rely on multidisciplinary approaches that include invasive exploration techniques in animal models. Mouse models offer substantial possibilities for genetic manipulation and have proven to be highly relevant for the understanding of human auditory defects because mutations in mouse orthologues of the genes associated with deafness in humans faithfully mimic the sensory defect in most cases.

**Fig. 2:**
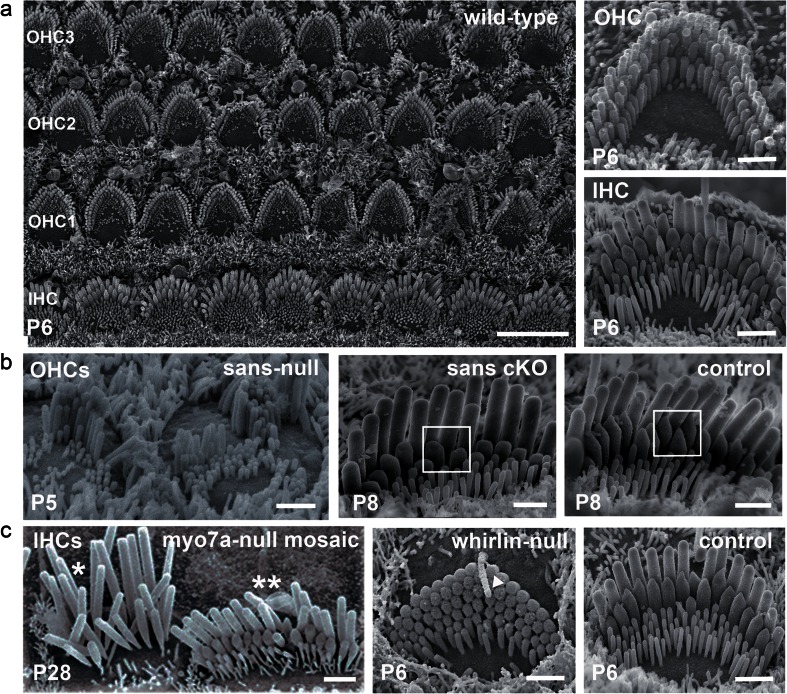
Hair bundle polarity and morphology. **a** *Left* Scanning electron micrograph (SEM) of the organ of Corti in a P6 wild-type mouse. The U- and V-shaped hair bundles of IHCs and OHCs are aligned and their vertices point towards the cochlear abneural edge. Scale bar, 5 μm. *Right* Examples of OHC and IHC hair bundles in a P6 wild-type mouse. Scale bars, 1 μm. **b** *Left* SEM of OHC hair bundles in a sans-null mutant mouse at P5 (Jackson shaker). *Right* SEM of a basal IHC hair bundle in a sans cKO (*Myo15-cre* ^*+/−x*^ *Ush1g* ^fl/fl^) mouse and in a control mouse at P8. The white frame highlights the presence and absence of prolate shapes of representative stereocilia tips for the control and the cKO genotype, respectively. Scale bars, 1 μm. **c** *Left* SEM of IHC hair bundles in a myo7a-null mosaic mouse mutant. In this mouse, *Myo7a* was expressed transgenically on the X-chromosome of myo7a-null mutants, enabling direct comparison, within the same organ of Corti, between myosin VIIa-deficient (*single asterisk*) and -complemented (*double asterisk*) hair cells due to random X-chromosome inactivation among hair cells [167]. Note that the stereocilia of the tallest row are longer in the myosin VIIa-deficient (*single asterisk*) IHC than in the myosin VIIa-complemented (*double asterisk*) IHC. *Right* SEM of an IHC hair bundle in a whirlin-null (whirler) mouse and in a control mouse. Note the abnormally short stereocilia in the whirlin-null IHC; as a result, the kinocilium (*arrowhead*) is taller than the stereocilia. Scale bars, 1 μm

The auditory sensory epithelium of mammals, which is called the organ of Corti (Fig. 1a), is comprised of the hair cells and of various types of supporting cells that are sandwiched between the underlying basilar membrane and the overlying tectorial membrane. Upon sound stimulation, the shearing movement between the basilar membrane and the tectorial membrane deflects the hair bundles of hair cells at the frequency of the stimulus. Each hair cell along the cochlear longitudinal axis is tuned to be highly sensitive to a particular frequency, called its characteristic frequency. Together, the hair cells form a tonotopic map from the base to the apex of the cochlea. There are two types of hair cells in the cochlea: the inner hair cells (IHCs), which are organised in one row, and the outer hair cells (OHCs), which are organised in three rows (Figs. 1a and 2a). IHCs are the genuine sensory cells that transduce the sound stimuli into an electrical signal in the primary auditory neurons, whereas OHCs carry out frequency dependent mechanical amplification of sound-evoked vibrations of the organ of Corti.

As more and more genes involved in MET are identified, a major challenge is to elucidate the physiological roles of the encoded proteins. More than 80 molecules have already been shown to be essential to MET (see Table 1). However, only a small proportion of these molecules have been identified as components of the MET machinery, based on electrophysiological data and relevant biophysical models. In particular, the molecular identity of the MET channel is still a matter of debate. The molecular motor myosin-VIIa was the first ‘deafness’ gene to be discovered [231, 72]; however, its role in auditory transduction and in particular, its role as a molecular conveyor and as a mechanical tensor has not yet been clarified. Some molecules play several roles at different positions in the hair bundle or at different stages in the development of the transduction apparatus [117, 35]. For instance, abnormal morphogenesis of the hair bundle in knock-out mice defective for such proteins may mask subsequent morphological or functional defects arising at late stages of development. Delayed conditional knock-outs in specific cochlear cell types are useful to examine the possible role of these molecules in the mature hair bundle [35, 160].

**Table 1:** List of molecules involved in MET

Gene	Protein	Human deafness form	Mouse mutant
Core PCP protein			
*VANGL2*	vangl2		*Vangl2* ^Lp^; *Vangl2Lp* ^cKO/cKO^
*VANGL1*	vangl1		*Vangl1* ^gt^
*FZD3*	frizzled-3		*Fz3* ^−/−^
*FZD6*	frizzled-6		*Fz6* ^−/−^
*DVL1*	disheveled-1		*Dvl1* ^−/−^
*DVL2*	disheveled-2		*Dvl2* ^−/−^
*DVL3*	disheveled-3		*Dvl3* ^cKO/cKO^
Non-core PCP protein			
*ROR2*	ror2		*Ror2* ^−/−^
*CTHRC1*	cthrc1		*Cthrc1* ^LacZ/LacZ^
*SCRIB*	scribble		*Scrib* ^Crc/Crc^
*PTK7*	PTK7		*Ptk7* ^Gt(Betageo)1Matl^
*FAT4*	fat4	Cystic kidney disease	*Fat4* ^−/−^
*DCHS1*	dchs1		*Dchs1* ^cKO/cKO^
*SEC24B*	sec24b		*Sec24b* ^Y613^
*SMURF1*	smurf1		*Smurf1* ^−/−^
*SMURF2*	smurf2		*Smurf2* ^−/−^
*GNAI3*	Ga_i3_ (Gnai3)		*Gai3* ^−/−^
*GPSM2*	GPSM2 (LGN)	DFNB82/Chudley-McCullough syndrome	*GPSM2* ^cKO/cKO^
*PRKCZ*	Prkcz (aPKC)		
Proteins involved in ciliopathies			
*BBS1*	BBS1	Bardet–Biedl form 1	*Bbs1* ^−/−^
*BBS4*	BBS4	Bardet–Biedl form 4	*Bbs4* ^−/−^
*MKKS*	BBS6	Bardet–Biedl form 6	*Mkks* ^*−/−*^
*TTC8*	BBS8	Bardet–Biedl form 8	*Bbs8* ^−/−^
*MKS1*	mks1	Meckel–Gruber syndrome	*Mks1* ^del64-323^
*ALMS1*	alms1	Alström syndrome	*Alms1* ^−/−^
*IFT88*	Ift88		*Ift88* ^cKO/cKO^
*KIF3A*	Kif3a		*Kif3a* ^cKO/cKO^
Cell-cell junction proteins			
*CLDN14*	claudin-14	DFNB29	*Cldn14* ^−/−^
*CLDN9*	claudin-9		*Cldn9* ^nmf329^
*CLDN6*	claudin-6		
*ZO1*	ZO-1		
*TJP2*	TJP2	DFNA51	
*VEZT*	vezatin		*Vezt* ^cKO/cKO^
Actin, actin-binding and actin-interacting proteins			
*ACTB*	β--actin	Deafness, dystonia	*Actb* ^cKO/cKO^
*ACTG1*	γ-actin	DFNA20/26	*Actg1* ^cKO/cKO^
*DIAPH1*	diaphanous-related formin 1	DFNA1	
*DIAPH3*	diaphanous-related formin 3	AUNA1	*Diap3* ^line771^ *; Diap3* ^line924^
*ESPN*	espin	DFNB36	Jerker (*je*)
*EPS8*	eps8	DFNBn	*Eps8* ^−/−^
*EPS8L2*	eps8L2		*Eps8L2* ^−/−^
*RDX*	radixin	DFNB24	*Rdx* ^−/−^
*TRIOBP*	TRIOBP	DFNB28	*Triobp* ^tm1Tbf^
*TPRN*	taperin	DFNB79	
*FSCN2*	fascin-2		B6.D2-*Fscn2* ^R109H^
*TWF2*	twinfilin-2		*Twf2* ^−/−^
*GSN*	gelsolin		*Gsn* ^tm1Djk^
Molecular motors			
*MYO1C*	myosin-Ic		*Myo1c* ^Y61G^
*MYO3A*	myosin-IIIa	DFNB30	*Myo3a* ^tm1.1Mckg^
*MYO3B*	myosin-IIIb		
*MYO6*	myosin-VI	DFNB37, DFNA22	Snell’s waltzer (*sv*); twist (*Twt*)
*MYO7A*	myosin-VIIa	DFNB2, DFNA11/USH1B	Shaker-1 (*sh1*); headbanger (*hdb*); *Myo7a* ^*6J*^ *; Myo7a* ^*4626SB*^
*MYO15A*	myosin-XV	DFNB3	Shaker-2 (*sh2*)
Hair bundle links and associated proteins			
*USH1C*	harmonin	DFNB18/USH1C	Deaf circler (*dfcr*); *Ush1c* ^−/−^
*CDH23*	cadherin-23	DFNB12/USH1D	Waltzer (*v*)
*PCDH15*	protocadherin-15	DFNB23/USH1F	Ames waltzer (*av*)
*USH1G*	sans	USH1G	Jackson shaker *(js), sans* ^cKO^
*USH2A*	usherin	USH2A	*Ush2a* ^*−/−*^
*GPR98*	VLGR1	USH2C	*Gpr98* ^del7TM^; *Gpr98* ^−/−^
*DFNB31*	whirlin	DFNB31/USH2D	Whirler (*wi*)
*PDZD7*	PDZD7		*Pdzd7* ^−/−^
*PTPRQ*	PTPRQ	DFNB84	*Ptprq* ^−/−^
*STRC*	stereocilin	DFNB16	*Strc* ^−/−^
*LHFPL5*	TMHS	DFNB66/67	Hurry-scurry (*hscy*); *THMS* ^−/−^
*TMC1*	TMC1	DFNB7/11, DFNA36	Deafness (*dn*); Beethoven (*bth*); *Tmc1* ^−/−^
*TMC2*	TMC2		*Tmc2* ^−/−^ *; Tmc2* ^tm1Lex^
Other stereociliary proteins			
*MPP5*	MAGUK p55		
*EPB41*	4.1R		
*CLRN1*	clarin-1	USH3A	*Clrn1* ^−/−^
*CIB2*	CIB2	DFNB48/USH1J	
*CLIC5*	CLIC5		Jitterbug (*jbg*)
*SLC9A3R1*	NHERF1		*Nherf1* ^−/−^
*SLC9A3R2*	NHERF2		*Nherf2* ^−/−^
*ATP2B2*	PMCA2		Deafwaddler (*dfw*); *Atp2b2* ^−/−^
*MAGI1*	MAGI1		
*TBC1D24*	TBC1D24	DFNB86	
*ELMOD1*	ELMOD1		roundabout (*rda*); roundabout-2J (*rda(2J)*)
*ELMOD3*	ELMOD3	DFNB88	
*LOXHD1*	LOXHD1	DFNB77	samba
*ATP8B1*	ATP8b1		*Atp8b1* ^G308V/G308V^
Tectorial membrane proteins			
*TECTA*	α-tectorin	DFNB21, DFNA8/12	*Tecta* ^DENT/DENT^ *; Tecta* ^Y1870^
*TECTB*	β-tectorin		*Tectb* ^−/−^
*OTOG*	otogelin	Overlaps DFNB18	*Otog* ^tm1Prs^
*OTOGL*	otogelin-like	Overlaps DFNB84	
*CEACAM16*	ceacam16	DFNA4	*Ceacam16* ^−/−^
*OTOL1*	otolin		
*OTOA*	otoancorin	DFNB22	*Otoancorin* ^−/−^
Proteins involved in K^+^ homeostasis			
*KCNJ10*	Kcnj10 (Kir4.1)		*Kcnj10* ^−/−^
*KCNQ1*	Kcnq1	Jervell and Lange–Nielsen syndrome	*Kcnq1* ^−/−^
*KCNE1*	Kcne1 (Isk)	Jervell and Lange–Nielsen syndrome	*Isk* ^−/−^
*NKCC1*	NKCC1 (Slc12a2)		*Slc12a2* ^−/−^
*GJB2*	connexin-26	DFNB1A/Vohwinkel syndrome	*Gjb2* ^cKO/cKO^
*KCNQ4*	Kcnq4	DFNA2A	*Kcnq4* ^dn/dn^ *; Kcnq4* ^−/−^
*SLC12A6*	KCC3	Anderman syndrome	*Kcc3* ^−/−^
*SLC12A7*	KCC4		*Kcc4* ^−/−^

For each entry, the name of the human gene, the associated protein, the corresponding forms of human deafness, and the main/historical mouse lines are listed (see references in the text). Several additional proteins with possible roles in MET have been included in the table. The membrane-associated guanylate kinase (MAGUK) protein MAGI1 was identified as a binding partner of cadherin-23. MAGI1 has a distribution similar to that of cadherin-23 in stereocilia and has been proposed to connect the MET machinery and the cytoskeleton [237]. Mutations in *TBC1D24*, previously known to cause epilepsy without reported hearing impairment, can also cause nonsyndromic deafness [246, 15, 169]. The function of the protein in hair cells is still unknown. The protein was detected in the hair bundle of P3 but not P7 OHCs, and patients were reported to have abnormal otoacoustic emissions, which indicate OHC dysfunction [246]. ELMOD1 and ELMOD 3 belong to the engulfment and cell motility (ELMO) protein family. Mutations in *ELMOD3* and *Elmod1* cause deafness in humans and mice, respectively [90, 93]. Both proteins have GTPase activating activity and could participate to actin dynamics in stereocilia through the Ras superfamily of small regulatory GTPases [90, 93]. Mutations in the ATP8b1 gene also cause deafness in humans and mice, and the encoded protein localises in stereocilia. In the mutant mice, sensory cells eventually degenerate, but the existence of normal ABR thresholds at P16 indicates that ATP8b1 would initially not be necessary for MET [203]. Mutations in the LOXHD1 gene cause progressive deafness and progressive degeneration of hair cells. The encoded protein has been detected in the developing and mature hair bundle [78]. DFNA and DFNB denote autosomal dominant and autosomal recessive isolated deafness forms, respectively

Any defect of the hair bundle is expected to have an effect on MET, including defects of hair bundle development, the tectorial membrane, which is involved in its deflection, the endocochlear fluid homeostasis, or the MET machinery itself. In this review, we examine knowledge gathered through neurogenetics regarding the molecules involved in these four aspects of hair bundle functioning, and discuss alternative strategies to complete the molecular picture of molecules involved in MET.

## Hair bundle development

### Positioning and orienting the hair bundle

Unlike humans that can detect sounds from the sixth month of embryonic development, mice start to hear on postnatal day 12 (P12) because their cochlear sensory epithelium continues to develop after birth. At birth, the first steps of hair bundle growth have already occurred. All the V- or U-shaped hair bundles are aligned, and their vertices point towards the cochlear abneural edge (see [131, 61] for review) (Fig. 2a). Planar polarisation of the hair bundles is essential for their coordinated deflection upon sound stimulation. Between embryonic day 14.5 (E14.5) and E15.5, a specialised primary cilium called the kinocilium, emerges at the centre of the hair cell apical surface, surrounded by microvilli, and migrates towards the cell’s abneural edge. Microvilli then grow differentially in a staircase pattern, eventually forming three stereocilia rows of increasing height. The position of the kinocilium marks the vertex of the hair bundle. Therefore, mutations in genes involved either in planar cell polarisation (PCP) or in kinocilium migration are expected to affect the final polarity of the hair bundle (see Table 1).

Core PCP molecules were originally identified from studies on *Drosophila. Vangl2* was the first orthologous gene to be implicated in the orientation of the hair bundle in the mouse [143]. *Vangl2* ^Lp/Lp^ mutants have normally shaped, but misoriented hair bundles. Defects in several other core PCP molecules including vangl1 [211], frizzled-3 [226], frizzled-6 [226], and disheveled-1, disheveled-2 and disheveled-3 [225, 58] also result in abnormally oriented hair bundles. These core PCP molecules are asymmetrically distributed within the cell and are mostly located at the junctions between hair cells and supporting cells. For example, vangl2 is highly abundant at the adherens junction on the supporting cell side [73, 227]. Mutations in non-core PCP genes including *Cthrc1* [239], *Ror2* [239], *Scrib* [143], *PTK7* [123, 158, 115], *Fat4* [180], *Dchs1* [128], *Sec24b* [139], *Smurf1* and *Smurf2* [148] also result in hair bundle misorientation. Mutations in these genes give rise to variable phenotypes that are usually less severe than those of mutations in the core PCP genes. Mutations in the genes causing ciliopathies, which are syndromes that result from defects of the primary cilium, also lead to defects of hair bundle polarity. They include some of the genes responsible for Bardet–Biedl syndrome (*BBS1* [175], *BBS4* [175], *MKKS* (*BBS6*) [175] and *TTC8* (*BBS8*) [132]) (see [66] for review), genes responsible for Meckel–Gruber syndrome (*Mks1* [44]), and genes responsible for Alström syndrome (*Alms1* [89]). The conditional knock-out of genes involved in intraflagellar transport, *Ift88* or *Kif3a*, results in loss of the kinocilium and is associated with PCP defects in mice, providing further evidence for the involvement of the kinocilium in hair bundle orientation [95, 200].

GTP-binding protein α_i_ subunits (Gα_i_) control mitotic spindle orientation and are associated with GPSM2, which is a protein implicated in deafness [221, 53]. Gα_i_ subunits were recently found to be involved in kinocilium migration and in hair bundle shape and orientation [60, 206]. These proteins are located in the apical region of the hair cell on its abneural side, between the cell junction and the hair bundle, forming a crescent-shaped domain. The role of Gα_i_ in hair bundle shape was confirmed in Gα_i3_ mutant mice that display flattened hair bundle shapes and mislocalised kinocilia [60]. A complementary domain to that of Gα_i_ at the apical surface of hair cells on the neural side of hair bundles is also defined by the expression of atypical protein kinase C (aPKC) [60, 206]. Thus, the boundary between the Gα_i_- and aPKC-containing areas may participate in defining the apical surface subregion where the hair bundle emerges [60, 206].

### The hair bundle, a cohesive structure

**Fig. 3:**
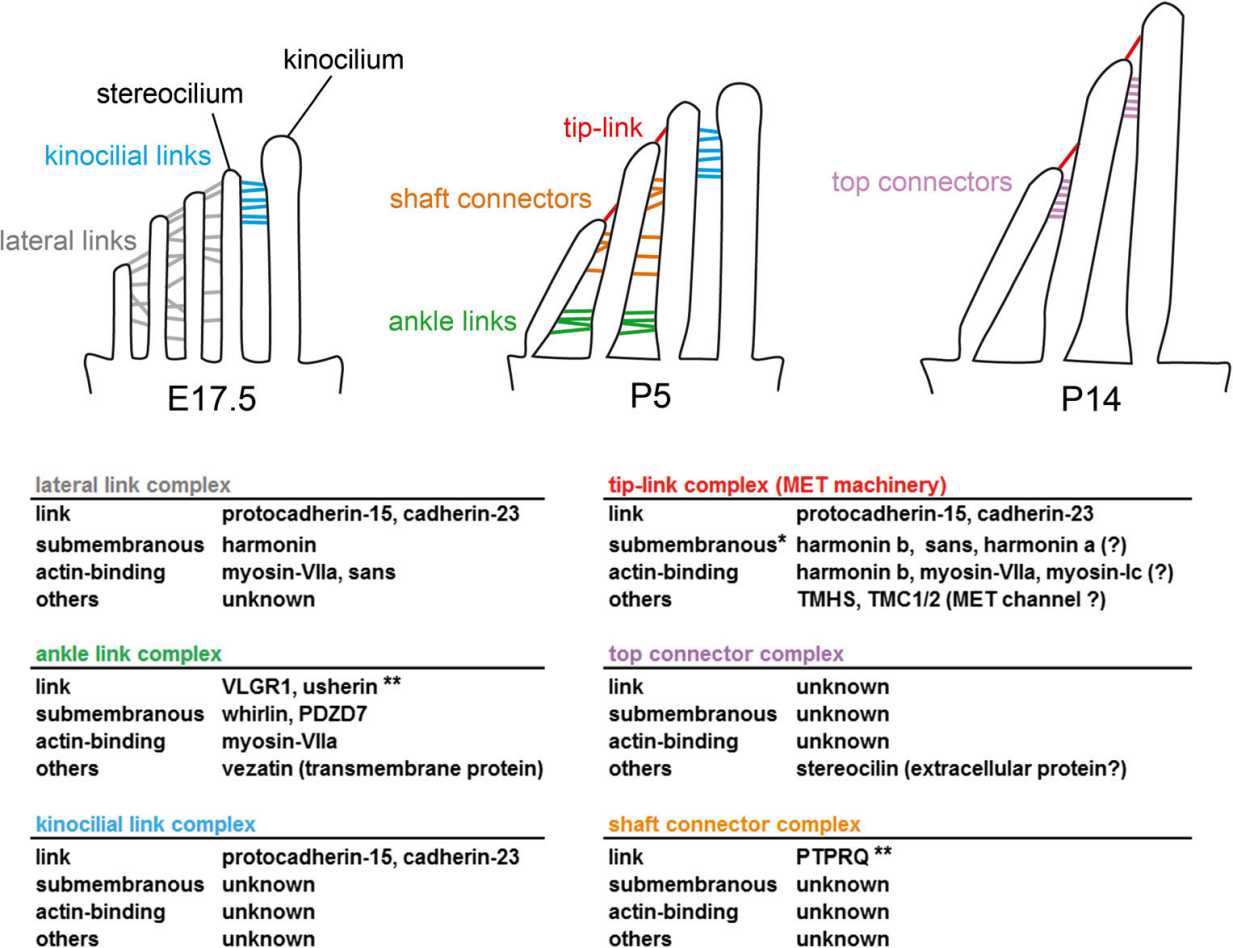
Hair bundle cohesion. *Top* Schematic illustration of the different types of links between stereocilia in OHCs at three different developmental stages, E17.5, P5, and P14. *Bottom* Molecular composition of the different links and their associated molecular complexes. *Single asterisk* The positions of the listed proteins at the upper or lower tip-link insertion points are detailed in Fig. 5. *Double asterisk* Usherin and PTPRQ are part of the ankle link complex and the shaft link complex, respectively, but it is unknown whether these proteins form the links

The formation of the hair bundle and the maintenance of its cohesiveness are orchestrated by several types of links that come into play at different developmental stages. Prior to their molecular description, these links were categorised according to both their location and sensitivity to proteases/calcium chelators (Fig. 3) [19, 75]. In the newborn mouse (P0), numerous interstereociliary lateral links interconnect stereocilia across and between rows in different directions. From P2 onwards, three types of lateral links take over, namely ankle links that are located at the base of stereocilia and shaft connectors that are located along stereocilia, and kinocilial links that connect the kinocilium to adjacent stereocilia of the tallest row. In mature cochlear hair cells, only the tip links remain, together with putative lateral links in IHCs and apical top connectors in OHCs [75]. Several molecular components of these links have been identified (see Table 1). Mutations in the corresponding genes in mice lead to congenital hearing impairment and hair-bundle disorganisation, indicating that each link type contributes critically to the building or the maintenance of the hair bundle.

The study of the genes responsible for Usher syndrome has been especially informative for our understanding of hair-bundle development. Usher syndrome (USH) is an autosomal recessive disorder that associates congenital hearing impairment with delayed onset retinitis pigmentosa eventually leading to blindness. This disorder has three clinical subtypes. USH1, the most severe form, is characterised by severe to profound congenital deafness, constant vestibular dysfunction and prepubertal onset retinitis pigmentosa. By contrast, USH2 involves only moderate to severe hearing impairment and no vestibular dysfunction. USH3 is distinguished from USH2 by the progressiveness of the hearing impairment and the occasional presence of vestibular dysfunction (see [163] for review). Six genes have been implicated in USH1, three in USH2 and one in USH3. USH1 has been associated with mutations in the genes encoding cadherin-23 (USH1D) [29, 31], protocadherin-15 (USH1F) [6, 8], the PDZ domain-containing protein harmonin (USH1C) [216, 25], the ankyrin repeat- and sterile α motif-containing protein sans (USH1G) [232] (Fig. 2b), the unconventional myosin myosin-VIIa (USH1B) [231] and the calcium and integrin-binding protein CIB2 (USH1J) [171]. USH2 has been associated with mutations in two genes encoding proteins containing a long extracellular domain, the very large G-coupled receptor (VLGR1) (USH2C) [233] and the transmembrane protein usherin (USH2A) [59], and with mutations in the gene encoding the PDZ domain-containing protein whirlin (USH2D) [57]. The gene encoding the four-transmembrane domain protein clarin-1 (USH3A) is the only identified gene associated with USH3 [91, 3, 64]. Genetics brought the first evidence that proteins involved in the various genetic forms of each Usher clinical subtype interact in vivo [26, 117]. In vitro binding experiments then demonstrated their direct interaction. These proteins are either components of the interstereociliary links or are submembrane scaffold proteins that presumably participate in the anchoring of these links to the actin cytoskeleton (Fig. 3). For instance, early transient lateral links, kinocilial links and tip links are made of cadherin-23 and protocadherin-15 [26, 75, 142, 198, 201, 5, 99]. Cadherin-23 forms a ternary complex with harmonin and myosin-VIIa [16]. Protocadherin-15 binds to myosin-VIIa [194] and binds to harmonin in vitro [2, 170]. Mutations in any of the mouse USH1 orthologous genes lead to cochlear hair bundle fragmentation, highlighting their role in hair bundle cohesion as early as E17 [109, 72, 234, 51, 7, 92, 104]. Moreover, the hair bundles of these mutant mice have mispositioned kinocilia and are misoriented [117]. Ankle links are composed of VLGR1 and possibly usherin [1, 136, 140]. These proteins interact with whirlin [214, 1] and PDZD7 [77, 250] that is encoded by a modifier gene of the USH2 phenotype [56]. In *Vlgr1* knock-out mice, ankle links are absent and abnormally shaped hair bundles are apparent at P2 [136, 238] (Fig. 3). Paradoxical MET currents can be elicited in these bundles if the stereocilia are deflected in the inhibitory direction by a glass pipette, indicating a lack of hair bundle cohesiveness [140]. In addition, two proteins that are implicated in isolated deafness but not in USH also play a role in hair bundle cohesiveness: tyrosine phosphatase receptor Q (PTPRQ) that is associated with shaft connectors [74, 149] and stereocilin that is associated with OHC top connectors [217, 218].

### Control of stereocilia length

Stereocilia are filled with a large core of parallel, densely packed, cross-linked actin filaments with barbed ends at their tips, where actin monomers are incorporated, and with pointed ends at their base, where depolymerisation occurs. Stereocilia taper at their base, which contains fewer actin filaments than the core. These filaments are densely packed to form an array that extends below the apical cell surface, forming the stereocilia rootlets. These rootlets anchor the stereocilia in the cuticular plate, which is a dense meshwork of actin filaments lying beneath the apical surface of the hair cell. The biophysical properties of MET strongly rely on the correct formation and maintenance of the hair bundle staircase pattern.

The shape of stereocilia reflects that of its cytoskeleton, which in turn depends on different categories of actin-interacting proteins. These include (1) actin-nucleating proteins that promote initiation of actin polymerisation, (2) actin-capping proteins that prevent the barbed end from incorporating actin monomers, (3) actin-bundling proteins that cross-link parallel actin filaments, (4) actin side-binding proteins, (5) actin-monomer-sequestering proteins, (6) actin-severing proteins that split actin filaments and (7) actin molecular motors. Mutations in various actin and actin-interacting proteins of these categories cause defects in stereocilia structure (see Table 1). Stereocilia contain β-actin (actb) and γ-actin (actg1), and mutations in *ACTG1* and *ACTB* lead to deafness [81, 144, 161, 166, 213, 249]. Mutations in *Diaphanous-1*, which encodes an actin-nucleating protein that controls actin polymerisation, cause deafness [125]. Overexpression of *Diaphanous-3* also results in deafness due to larger than normal stereocilia [189]. Espin, an actin-bundling protein, is necessary for the assembly and stabilisation of parallel actin filaments. Stereocilia morphogenesis is markedly impaired in the Jerker mutant mouse, which lacks functional espin [248, 150]: as early as P0, stereocilia are abnormally thin and short, with impaired differential elongation that causes the loss of the staircase pattern [191]. Mutations in *EPS8*, which encodes an actin-bundling and actin-capping protein, cause profound congenital deafness [20]. Eps8 is located predominantly at the tips of stereocilia. In knock-out mice lacking eps8, stereocilia are abnormally short but are still organised in a staircase pattern [244]. Notably, a related actin-bundling and actin-capping protein, eps8-l2, is required for the maintenance of the hair bundle staircase pattern [68]. Radixin (rdx), which belongs to the family of ezrin/radixin/moesin (ERM) proteins, tethers actin filaments to the plasma membrane at the base of stereocilia. Accordingly, mutations in *RDX* are responsible for hearing impairment in humans [101], and loss of *Rdx* in mice causes progressive degeneration of stereocilia [107]. NHERF1 and NHERF2, which both contain an ERM binding domain and two PDZ domains [54], have also been implicated in deafness in mice [96]. NHERF2 is mainly located at the base of hair bundles of cochlear hair cells and is more abundant in IHCs than in OHCs [196, 96]. NHERF1 is present in the hair bundles of both IHCs and OHCs at embryonic stages before concentrating at the stereocilia tips of OHCs and could possibly bind to cadherin-23 in vivo [96]. In *Nherf1* ^−/−^ mice, the hair bundles of OHCs have abnormal shapes in the basal and middle cochlear regions. Interestingly, this tonotopy-dependent phenotype has revealed an unusually powerful mode of interference between low- and high-frequency sounds, suggesting a previously unreported mode of off-frequency hearing [96]. Studies involving *Triobp* mutant mice, which lack both TRIOBP-4 and TRIOBP-5, show that the actin-bundling protein TRIOBP is necessary for the formation of stereocilia rootlets [108]. Many other actin-interacting proteins have been detected in stereocilia including the actin side-binding protein tropomyosin [69], the actin-severing protein cofilin [146] and the actin-bundling proteins fimbrin [210] and fascin-2 [162].

**Fig. 4:**
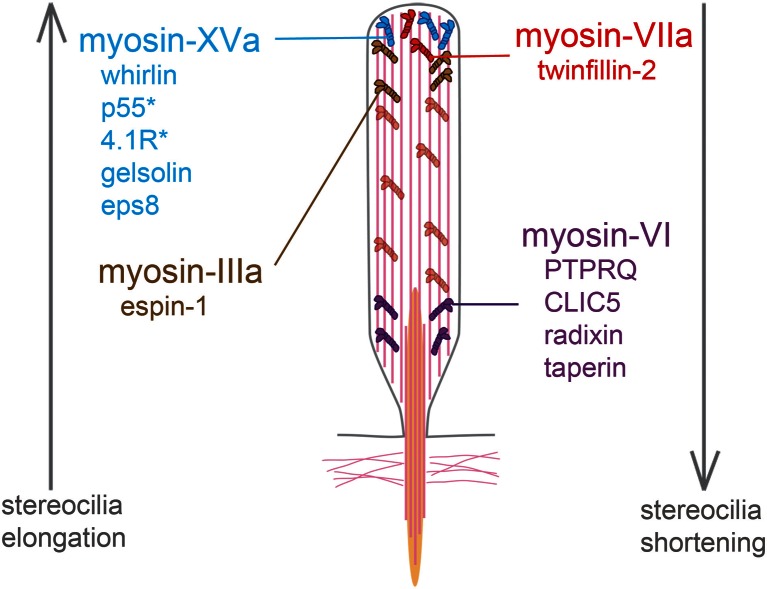
List of myosins and their interactors involved in the control of stereocilia length. The roles of myosin-VI, myosin-VIIa, and myosin-XV have been determined by the study of mutant mice defective for these proteins. In contrast, the implication of myosin-IIIa in stereocilia elongation was assessed in vitro from the observation that stereocilia are taller than normal in co-transfected hair cells producing myosin-IIIa and espin 1 [184]. *Single asterisk* These proteins have not been associated with deafness forms in humans or in mice

The hair bundle also contains various unconventional myosins. Their respective contributions in molecular transport and in the maintenance of mechanical tension have not yet been clarified. Myosins are logical candidates to transport proteins along the stereocilia dense network of actin filaments [209]. Moreover, their presence at different locations, especially near the tip or at the base of stereocilia, may exert tension on actin filaments and modify stereocilia shape. The study of myosin-IIIa, myosin-VI, myosin-VIIa and myosin-XV has provided additional information about the molecular complexes involved in the maintenance of the stereocilia actin cores (Fig. 4). Myosin-IIIa [222] accumulates at stereocilia tips [188, 223] and promotes stereocilia lengthening when overexpressed with espin-1 in hair cells [184]. Stereocilia grow excessively and fuse together in mutant mice deficient for myosin-VI [14, 13, 193]. It has recently been proposed that myosin-VI participates in a molecular complex with CLIC5, PTPRQ, radixin and taperin, which are all present at the base of stereocilia [183, 70]. This complex may help to stabilise interactions between the plasma membrane and the subcortical actin cytoskeleton, which may explain the fusion of stereocilia in myosin-VI-deficient mice [182, 183]. Nonetheless, the mechanism of stereocilia overgrowth in these mice is still poorly understood. The tallest row of stereocilia in mutant mice deficient for myosin-VIIa is also abnormally long [167] (Fig. 2c). This phenotype has been ascribed to the concomitant loss of twinfilin-2, an actin-sequestering and actin-capping protein that inhibits actin polymerisation [178, 159]. Another molecular complex was uncovered by the observation of abnormally short stereocilia in myosin-XV-defective [165] and whirlin-defective mouse mutants [134] (Fig. 2c). Myosin-XV and whirlin interact and form a complex with eps8 that plays a crucial role in the elongation of the stereocilia actin filaments [50, 21, 127]. This complex also includes the membrane-associated guanylate kinase (MAGUK) p55, protein 4.1R [133] and gelsolin, which is an actin-capping and actin-severing protein [135]. Therefore, several myosin-dependent molecular complexes that are linked to actin dynamics work in concert to determine stereocilia length.

The molecular processes that determine stereocilia differential elongation in different rows are still unknown. However, several studies, with conflicting results, have addressed the issue of steady-state actin renewal in mature hair bundles. A treadmilling process was first proposed to ensure the renewal of actin monomers in stereocilia filaments. When actin fused to the green fluorescent protein (actin-GFP) was overexpressed in cells, the actin core renewal speed was unexpectedly fast (~48 h) [187], and turnover time was similar in different stereocilia rows. This implies an approximate proportional relationship between stereocilia size and the speed of actin polymerisation [179]. However, the overexpression of a modified actin monomer (actin-GFP) might alter the intrinsic properties of actin in stereocilia. An alternative approach based on the incorporation of ^15^N-labelled precursor amino acids by multi-isotope imaging mass spectrometry in stereocilia indeed suggested otherwise, i.e. that the overall protein renewal including actin is slow (around 10 days in young mice and 50 days in adult mice) and faster at the very tip (distal 0.5 μm end) than in the core of stereocilia [245]. However, the time resolution in this radio-labelling approach is limited by the life time of proteins, which might be much longer than the local turnover time of actin filaments by a treadmilling process.

## The mature MET apparatus

### The MET machinery

High-speed imaging of the calcium influx through MET channels in cochlear hair cells has shown that these channels are located at the tips of the short and middle row stereocilia but not in tall row stereocilia. MET channels would therefore be located at the basal ends of the tip links [24]. The molecular nature of the MET channel has so far remained elusive. The transmembrane channel-like 1 (TMC1) and TMC2 proteins, which have six transmembrane domains, are currently the best candidates. Indeed, mutations in *TMC1* cause deafness in humans [113] and inner ear hair cells from double knock-out mice for *Tmc1* and *Tmc2* have no MET currents [98]. In addition, the re-expression of various combinations of *Tmc1*, *Tmc2*, and mutated forms of *Tmc1* in the hair cells of these double knock-out mice [157] modifies the single MET channel conductance and its permeability to Ca^2+^ ions. This suggests that TMC1 and TMC2 are pore-forming subunits of the MET channel [157, 106]. However, this view was recently challenged by the observation that a mechano-sensitive current could still be elicited in the double knock-out mice by pushing the hair bundle in the inhibitory direction [105]. Therefore, TMC1 and TMC2 may not constitute the MET channel by themselves, but instead may be essential for its targeting to the stereocilia tips (see [18] for comment and see [83] for review). A recent study revived the debate by showing that the ion channels underlying the anomalous MET current elicited by pushing the hair bundle in the inhibitory direction may in fact have pore properties different from those of the genuine MET channels, based on the lower dihydrostreptomycin-blocking efficacy and the absence of rectification in their current–voltage relationship [129].

**Fig. 5:**
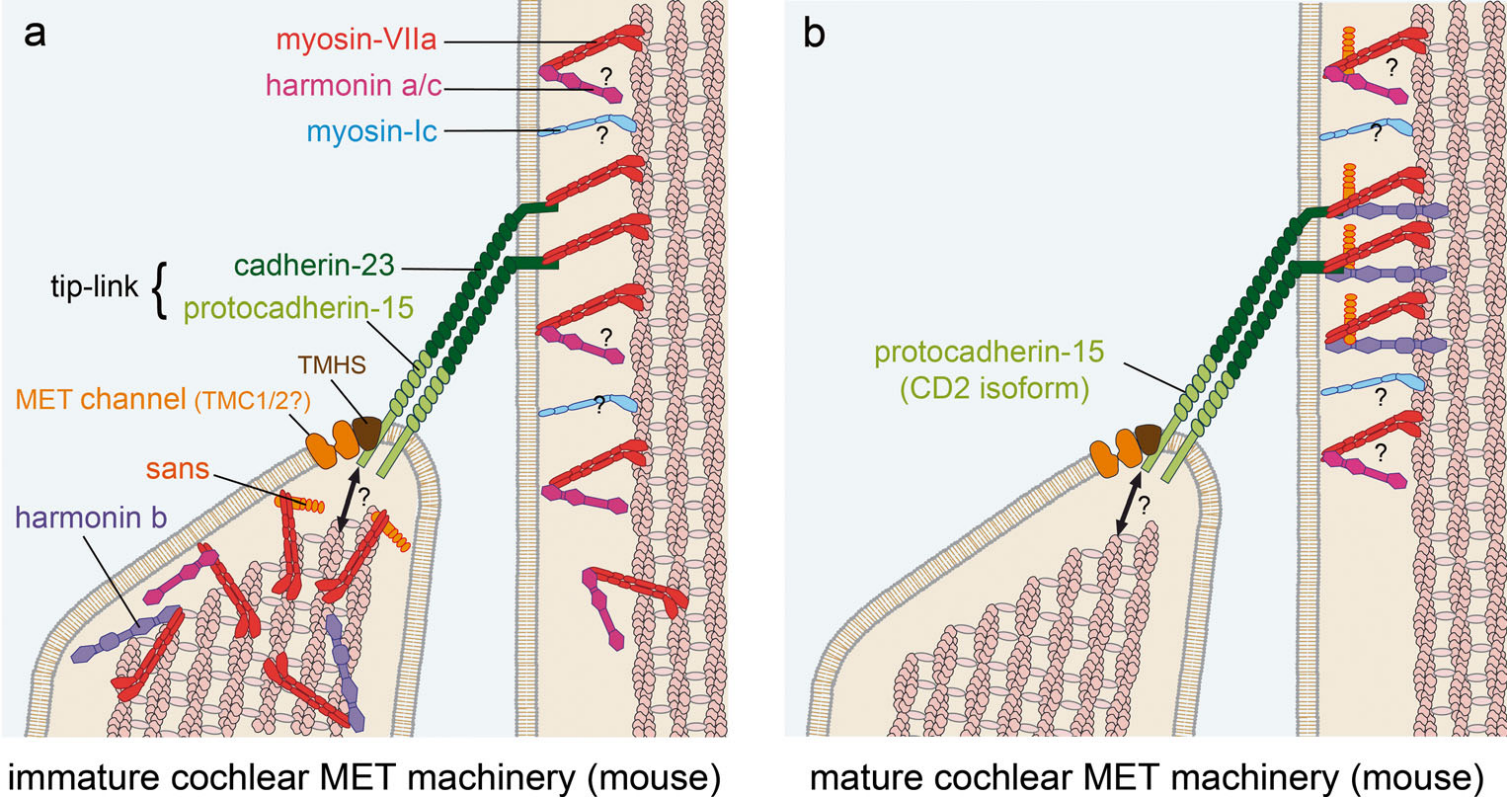
The MET machinery in cochlear hair cells. **a** In the developing hair bundle, the MET machinery comprises the MET channel(s) and TMHS at the lower tip-link insertion point. Sans and myosin-VIIa are also present, but the nature of their interaction with the MET complex is still unknown. The nature of the interaction between the MET complex and the actin cytoskeleton is also unknown at the lower tip link insertion point. At the upper tip-link insertion point, myosin-VIIa and harmonin b interact with cadherin-23. The role of myosin-Ic remains unclear in the cochlea because its function has not yet been tested in mice mutant for this protein. In addition, the location of myosin-Ic cannot be investigated by immunohistochemistry due to the absence of the appropriate mutant mice to confirm the specificity of antibodies directed against this protein. **b** Mature MET machinery. Sans, myosin-VIIa and harmonin-b are located at the upper tip-link insertion point

The upper and lower parts of the tip link are composed of cadherin-23 (USH1D) and protocadherin-15 (USH1F), respectively [198, 201, 5, 99]. Inner ear hair cells express three different transmembrane protocadherin-15 isoforms, CD1, CD2 and CD3, that differ in their intracellular amino acid sequence [5]. Based on the study of knock-out mice, each of them being defective for only one protocadherin-15 isoform, it has been suggested that protocadherin15 isoforms are functionally redundant [230]. However, the analysis of a delayed, hair cell-specific conditional knockout mouse that loses only the protocadherin-15-CD2 isoform after the period of hair-bundle development has shown that this isoform is an essential component of the tip link in mature auditory hair cells [160]. In addition, a *PCDH15* mutation that affects only the CD2 isoform was also found to lead to profound deafness without vestibular defects in human patients. Because mutant mice for CD1 or CD3 are not hearing-impaired [230], CD2 would be the only isoform of protocadherin-15 required for the tip link in mature IHCs and OHCs, unless CD1 and CD3 are functionally redundant [160]. Three other USH1 proteins, harmonin, sans and myosin-VIIa, and a non-USH gene, *tetraspan membrane protein of hair cell stereocilia* (*TMHS*), have been shown to participate in molecular complexes associated with the lower and upper tip-link insertion points (Fig. 5). Harmonin isoforms comprise three sub-classes: a, b, and c. The largest isoform, harmonin-b, that contains three PDZ domains, two coiled-coil domains and one PST domain, is an F-actin-bundling protein [26] and is located at the upper tip-link insertion point in the mature hair bundle [117, 141, 79]. Electrophysiological studies of MET currents in cochlear explants of harmonin-b null mice are consistent with a role of this protein as an internal linker between the tip link and the actin cytoskeleton [141]. The contributions of isoforms a and c to MET are still unclear [26, 79, 141]. Sans, which binds to harmonin [2, 240] and myosin-VIIa in vitro [2, 235], and possibly to the intracellular domains of cadherin-23 and protocadherin-15, is located at the lower tip-link insertion point in the developing hair bundle [35] and at the upper tip-link insertion point in the mature hair bundle [76]. Late conditional knock-out (after the development of the hair bundle) of the sans gene in hair cells results in markedly impaired transduction currents [35]. This has been ascribed to the loss of the tip links, implying that sans is necessary to maintain the tip link in the mature MET machinery. The involvement of myosin-VIIa in MET is likely to be more complex than that of sans since this motor protein probably has several functions. Mutant mice defective for myosin-VIIa have severely damaged hair bundles [192]. This myosin interacts with most of the other USH proteins and may intervene in their transport in the hair bundle, which may explain this phenotype. For instance, in myosin-VIIa-defective mouse mutants, two major components of the ankle-link complex, VLGR1 and usherin, are absent from the hair bundle as well as harmonin-b, but not cadherin-23 [140, 117, 194]. In the mature hair bundle, myosin-VIIa is observed in the region of the upper tip-link insertion point [76], where it is expected to form a ternary complex with harmonin-b and cadherin-23 as it does in vitro [16]. All USH1 proteins identified so far are involved in the MET machinery (Fig. 5), although the role of CIB2 has not yet been defined [171]. Finally, *TMHS*, a non-USH gene responsible for an autosomal recessive form of deafness, encodes a four-transmembrane domain protein that is located at the lower tip-link insertion point. TMHS binds to protocadherin-15 in vitro. *Tmhs* knock-out mice have very weak MET currents. However, this phenotype is partially rescued by the overexpression of protocadherin-15, indicating that impaired MET was mostly due to the defective recruitment of this protein. This suggests that TMHS is a key component of the MET machinery, possibly bridging protocadherin-15 to the MET channel, but is not a component of the MET channel itself [236] (Fig. 5).

### The MET machinery, a structure under tension

Several features indicate that the MET machinery is subjected to tension even in the absence of sound stimuli. Stereocilia tips of short and middle rows have a prolate shape that is thought to be caused by the resting tension exerted by the tip link on the plasma membrane (Fig. 2b). Direct recordings of receptor potentials in cochlear hair cells in response to sound stimulation in vivo, and of MET currents in response to displacement of the hair bundle in vitro, have shown that a proportion of MET channels are open at rest [42, 45, 176, 94]. This suggests that the resting tension applied to the MET machinery is tightly controlled. This tension is perturbed in several mouse mutants involving molecules of the MET machinery. The phenotypic consequences of conditional knock-out of the sans gene appear at P8 and involve the simultaneous loss of tip links and of the prolate shape of IHC stereocilia tips (Fig. 2b). The prolate shape of stereocilia tips is also absent in cadherin-23 conditional knock-out mice that display an abnormal phenotype involving mature hair cells (beyond P23). Interestingly, in these two models, the loss of the prolate shape is concomitant with the regression of stereocilia in the short and middle rows [35, 34]. These observations are consistent with the hypothesis that tip-link tension controls actin polymerisation at the barbed end of stereocilia actin filaments [168].

The control of the holding tension on the MET machinery depends on the anchoring of the MET channel and the tip link to the actin cytoskeleton. The tip-link tension can be modulated by sliding of the tip-link upper end anchoring point along actin filaments. This mechanism is thought to contribute to the adaptation process that is reflected in the decline in the transduction current evoked by a step displacement of the hair bundle in vitro [85, 9, 55, 111]. Myosins, which are actin-based motors, are natural candidates for the control of tip-link tension by this mechanism. A chemical–genetic strategy in the mouse indeed provided support for a critical role of myosin-Ic in the MET adaptation process in vestibular hair cells [82, 204]. However, it remains unclear which myosin(s) are involved in cochlear hair cells. Myosin-VIIa, which is present at the tip-link upper insertion point in mature cochlear hair cells, is a promising candidate for the MET machinery. However, the role(s) of myosin-VIIa in MET remain(s) unclear because hair bundles in the mutant mice defective for myosin-VIIa are strongly disorganised, making it difficult to attribute the abnormal functional features to a malfunctioning of the MET machinery only. Moreover, MET currents observed in *Myo7a* ^*4626S*B^ mice show characteristics similar to the abnormal currents observed in TMC1 and TMC2 defective mutants when hair bundles are pushed in the inhibitory direction, which suggests that the recorded MET currents in *Myo7a* ^*4626S*B^ mice would not be gated by tip links (see above) [110, 129]. The b isoform of harmonin also participates in the anchoring of the tip-link upper end to the actin cytoskeleton. In mutant mice that only lack this isoform, MET currents display a variable extent of adaptation. This observation is consistent with a role of harmonin-b as a component of the “extent spring” [195], a mechanical element that has been postulated to control the stroke of the myosin motors in the adaptation process [141]. The dynamic interplay between myosin-VIIa and harmonin-b, both of which can bind to actin at the upper tip-link end, still has to be elucidated. At the lower tip-link insertion point, little is known about the molecules that anchor the MET machinery to the actin cytoskeleton, even though several myosins are present at the stereocilia tips, including myosin-IIIa, myosin-IIIb [138] and myosin-XV (see above).

### The tectorial membrane

In the cochlea, hair bundles are covered by an acellular gel composed of several types of collagen and non-collagenous glycoproteins called the tectorial membrane. Like the organ of Corti, the tectorial membrane runs along the cochlear duct. It is attached on its medial side to the spiral limbus, and on the other side, it is in firm contact with the tips of the tallest OHC stereocilia row. Notably, hair bundles of IHCs are free standing under the tectorial membrane. Upon sound stimulation, the shear movement between the basilar membrane and the tectorial membrane drives hair bundle oscillations. Many proteins involved in the composition of the tectorial membrane or required for its attachment to hair cells are encoded by genes associated with deafness. The study of mice mutant for these genes has shed new light on the different roles played by the tectorial membrane in auditory MET.

Six non-collagenous glycoproteins have been found in the tectorial membrane: α-tectorin, β-tectorin, otogelin, otogelin-like, CEACAM16, and otolin [173, 121, 37, 199, 208, 247, 243, 30, 46, 97] (see Table 1 for deafness genes). Notably, the targeted mutation of α- and β-tectorin in mice has helped to characterise the mechanical properties of the tectorial membrane. The bulk of the tectorial membrane is made of several collagen fibres that are organised into a matrix composed of α- and β-tectorins. Inactivation of the α-tectorin gene in *Tecta* ^*ΔENT/ΔENT*^ mice, which causes the tectorial membrane to detach from the surface of the organ of Corti, led to the conclusion that the elasticity of the tectorial membrane has little influence on the amplitude and phase of deflexion of OHC stereocilia at the characteristic frequency. Rather, at this frequency, the tectorial membrane probably behaves mostly as an inert mass on which OHC stereocilia can react, ensuring that the OHCs respond to sound stimulation with the proper gain and timing [119]. Subsequently, the study of a knock-in mouse harbouring the semi-dominant *Tecta* Y1870C mutation pinpointed a second mechanical role of the tectorial membrane. Although OHC MET activity is normal in *Tecta* ^Y1870C/+^mice, neural thresholds are markedly high, indicating that the tectorial membrane also plays a critical role in driving the hair bundles of IHCs [120]. Three knock-in mouse lines with different missense mutations that change amino acid residues in distinct protein subdomains of α-tectorin have recently been produced. The analysis of these mice showed that these subdomains, when defective, affect the biomechanical properties of the tectorial membrane in different ways [118]. A third mechanical role has also been attributed to the tectorial membrane. The striated sheet formed by the two tectorins is disrupted in knock-out mice for the β-tectorin gene (*Tectb* ^*−/−*^ mice). Basilar membrane and neural tunings are both sharper than normal in these mice, suggesting that the tectorial membrane also influences the longitudinal spread of sound-induced excitation along the cochlea [177]. Several molecules involved in the two main attachments of the tectorial membrane have also been characterised. Otoancorin, which is present at the apical surface of the spiral limbus, plays a critical role in the attachment of the tectorial membrane to this structure. In otoancorin knock-out mice, the tectorial membrane is still attached to the OHC stereocilia but detaches from the spiral limbus, leading to the defective stimulation of IHCs [124]. Notably, the OHC response in these mutants is largely unaffected, despite the concomitant detachment of the TM from the spiral limbus. This reinforces the hypothesis that the elasticity of the tectorial membrane plays little role in the stimulation of OHCs near their characteristic frequency. Stereocilin is an extracellular protein of the mature OHC hair bundle. Top connectors do not form in stereocilin knock-out mice, and stereocilia imprints do not appear on the tectorial membrane. Thus, stereocilin is necessary for the formation of top connectors, and it may be a component of the “attachment links” that connect the tallest stereocilia of OHCs to the tectorial membrane. Whether these attachment structures are formed by genuine fibrous links or by the extracellular matrix remains unclear. The absence of the top connectors leads to deafness caused by progressive disorganisation of the hair bundle, which is preceded by a loss of the acoustic distortion products normally generated by OHC hair bundles [218, 215] (see [11] for review).

### Ionic composition of the endolymph

Hair bundles are bathed in endolymph, which is an extracellular fluid with an unusually high K^+^ concentration (approximately 150 mM [185]). There is a +80–100 mV transepithelial potential difference between the endolymphatic and perilymphatic compartments (endocochlear potential) [137, 153, 186]. The resulting 120–150 mV difference between the endolymph and the intracellular compartment [94] drives the MET current, mainly carried by K^+^ ions, into the hair cells. The endocochlear potential and the high K^+^ concentration of the endolymph are produced by the stria vascularis, a specialised bi-layered epithelium of the cochlear duct outer wall. The maintenance of the endocochlear potential requires the integrity of the cell–cell tight junctions that keep the endolymphatic and perilymphatic compartments electrically isolated from one another. Several ion channels and transporters have been implicated in the production of the endocochlear potential and/or K^+^ secretion by the stria vascularis, including the Kcnj10 [130, 242], Kcnq1 [151, 116], and Kcne1 [219, 212, 190] K^+^ channel subunits, and the Na^+^–K^+^–2Cl^−^ cotransporter NKCC1 [49, 52]. Loss-of-function mutations in any of these genes result in severe hearing impairment.

The existence of a recycling, through an intercellular gap junction network, K^+^ ions that flow out of the hair cells in their basolateral region has been suggested, although such a process remains to be established. Mutations in the connexin 26 gene (*CX26/GJB2*) [100] are the most common cause of autosomal recessive congenital deafness in many Caucasian populations; however, the various roles of gap junction channels in the functioning of the cochlea are still poorly understood. The conditional knock-out of *Gjb2* in the mouse organ of Corti leads to the degeneration of sensory cells and supporting cells. This phenotype has been attributed to defects in the gap junctions that would be involved in the recycling of K^+^ ions released at the base of hair cells. In addition, the endocochlear potential builds up but fails to be maintained in these mice, probably as a consequence of the loss of tight junctions between hair cells and their supporting cells [38]. The connexin 30 gene (*CX30/GJB6*) is contiguous with *CX26/GJB2* on human chromosome 13 (mouse chromosome 14) and is also expressed in the cochlea [207, 65]. Deletions in *GJB6* have been reported in deaf patients [122, 48, 156, 47]. Observations made from the first *Gjb6* knock-out mouse model led to the mistaken conclusion that inactivation of *Gjb6* alone could lead to deafness [207]. In fact, inactivation of the *Gjb6* gene, both in humans and in mice, also impaired the expression of the *Gjb2* gene [40, 174, 155, 126], and transgenic expression of *Gjb2* in the same *Gjb6* knock-out mouse model restored hearing [4]. Indeed, auditory brainstem responses were normal in a second, more recent *Gjb6* knock-out mouse mutant, in which sufficient expression of *Gjb2* was preserved. Thus, the cause of deafness after *GJB6* deletion is the low expression of *GJB2* due to the co-deletion of its putative regulatory element [39, 32]. In addition, the endocochlear potential in the first *Gjb6* knock-out mouse model [207] fails to build up as a consequence of abnormal tight junctions between endothelial cells in capillaries of the stria vascularis [32] indicating a role of Gjb2 at this emplacement. At least, three other genes are thought to be involved in the recycling circuit of K^+^ ions: *KCNQ4* [112], *KCC3* [28], and *KCC4* [27]. *KCNQ4* encodes a K^+^ channel subunit and *KCC3* and *KCC4* encode K^+^–Cl^−^ cotransporters. Kcnq4 is located at the base of mature OHCs and mediates a voltage-activated K^+^ current that is already active at the resting membrane potential [84, 103]. In *Kcnq4* ^*−/−*^ mice, this current is abolished, leading to a slow degeneration of OHCs, which probably results from their chronic depolarisation [102]. Kcc3 and Kcc4 are present in the supporting cells of IHCs and OHCs. Kcc3 and Kcc4 are thought to siphon K^+^ ions from the hair cells’ pericellular space into supporting cells, where these ions would enter the gap junction recycling pathway. Hair cells undergo degeneration both in *Kcc3* knock-out mice and *Kcc4* knock-out mice, although degeneration occurs earlier in the former than in the latter [27, 28].

The maintenance of the high endolymphatic K^+^ concentration and of the endocochlear potential requires strong apical cell–cell junctions in the epithelia lining the endolymphatic compartment of the cochlea, especially in the mechanically stressed sensory epithelium. Junctions between OHCs and their supporting cells are probably subjected to the highest amount of mechanical stress, due to the motion of the sensory epithelium and forces generated by OHC electromotility. These junctions are composed of an atypical combination of tight junctions and adherens junctions [152] containing claudin-14, claudin-9, claudin-6, catenins, ZO-1, TJP2 and vezatin [22, 147, 17, 220] (see Table 1 for deafness genes). This atypical junction complex probably plays a major role in the resilience of these cell junctions to mechanical stress. Indeed, conditional mutant mice deficient for vezatin in OHCs suffer from late onset hearing loss that can also be induced irreversibly by exposure to loud sound levels that are harmless to control mice [17].

## Continuing the molecular deciphering of the MET apparatus

There has been for the past 10 years remarkable progress in the identification of proteins and protein complexes that constitute the MET machinery. However, the composition of the central element of this machinery, the MET channel, is still under debate. Various strategies to characterise the molecular identity of this channel have been hindered by the limited amount of available material, the multifunction of particular molecules in the developing and mature hair bundle and by the current inability to reconstitute the MET machinery in a controlled exogenous system (see [145] for review). Genetic studies, both in humans and in mice, circumvented the problem of the paucity of the hair cell material available. The development of new genetic tools in the mouse, such as the myosin-XV promoter-driven *cre* mouse that enables delayed conditional knocking-out of proteins, offers a unique opportunity to distinguish the role of a particular protein in the mature hair bundle from its possible role during development [35, 160] (Fig. 2b). Other *cre* knock-in lines need to be developed to offer a larger panel of genetic tools at different developmental time points and in specific hair cell types. Studies that apply the same strategy to known components of the MET machinery should clarify their respective roles in the mature hair bundle.

Most genes that have been associated with deafness appear to affect MET either directly or indirectly. It is likely that the genetic approach will continue to feed the list of molecules involved in MET. As time passes, the increasing speed and smaller cost of exome sequencing will probably compensate the lower probability of finding new disease-associated loci by genetic linkage analysis of affected families. All USH1 proteins characterised so far have been implicated in the MET machinery; therefore, we can anticipate that the last USH1 protein identified, CIB2 (USH1J), will be no exception [171].

The retina is also affected by USH. The search for new binding partners of USH1 proteins in the retina is facilitated by the abundance of photoreceptor cells and may help to find new elements of the cochlear MET machinery. Until recently, the pathogenesis of the retinitis pigmentosa observed in USH1 patients remained elusive because mouse models for USH1 genetic forms do not reproduce the retinal degeneration phenotype of humans. The study of USH1 protein distribution in the macaque retina revealed the structural origin of this discrepancy [181]. In primate photoreceptor cells, USH1 proteins are present at the interface between inner and outer segments and are also associated to calyceal processes [33], which are axially oriented microvillus-like structures that form a collar around the base of the outer segment in rod and cone photoreceptors. Strikingly, calyceal processes are absent from the photoreceptor cells of mice, which probably explains the absence of an abnormal retinal phenotype in USH1 mutant mice. Calyceal processes resemble cochlear stereocilia in many respects. USH1 proteins are present in these structures, together with other molecules of the cochlear hair bundle such as myosin IIIa, espin, and the Ca^2+^ pump PMCA2 (plasma membrane calcium ATPase 2), which has also been implicated in mouse and human deafness [63, 202]. Furthermore, both cadherin-23 and protocadherin-15 are located at the membrane interface between the outer segment and surrounding calyceal processes and between the base of the outer segment and the apical region of the inner segment. The USH1 protein complex may form an adhesion belt connecting the outer segment basal region to the surrounding structures. These similarities between calyceal processes and hair cell stereocilia indicate that the study of photoreceptors may provide an alternative strategy to decipher the molecular elements of the MET machinery [181].

Human genetics has uncovered numerous molecules involved in hair bundle development and function. Each of these molecules provides a starting point to decipher whole molecular complexes. Clearly, the probability of finding new genes associated with deafness in patients from newly recruited families decreases with time, and as a consequence, this approach may cease to provide new candidates at some point. Moreover, lethal mutations cannot be detected by the human genetics approach, which may make some essential components of the MET machinery difficult to identify with this approach. Thus, complementary strategies need to be developed to complete the picture of the molecular networks in the hair bundle. In addition to the yeast two-hybrid technique that can find new interacting components of a molecular complex step by step [114, 133, 237], recent technological leaps have offered new screening strategies. Analysis of isolated hair bundles by mass spectroscopy could establish an extensive list of hair bundle proteins and their relative abundances, which would provide a new framework to pursue functional studies. Among the most abundant proteins, many are involved in the organisation of the actin cytoskeleton, in the maintenance of local ATP levels (the brain isoform of creatine kinase) [197, 196, 12], in calcium homeostasis (calcium buffering proteins such as parvalbumin, calbindin and calmodulin [80, 197], and the Ca^2+^ pump PMCA2 [63, 202]). Likewise, next generation sequencing coupled with messenger RNA amplification of a few sensory hair cells should bring new insight into the molecular components involved in hair cell MET. The variety of structures in which these components are involved implies that the understanding of their functions will rely more and more on in vivo studies in the future. Genetically modified mice have proven to be a powerful tool to study the role of molecules in situ. In addition, the replication of relevant human point mutations in mice has been very instructive, as illustrated by the use of particular *Tecta* and *Tectb* mutations to uncover the various roles of the tectorial membrane in MET. This mutational approach is to be extended with the arrival of more powerful and faster tools to engineer mouse mutants, such as the clustered regularly interspaced short palindromic repeat/CRISPR-associated (CRISPR/Cas) system to perform genome sequence specific-editing. The CRISPR/Cas system allows the one-step generation of mice carrying mutations in several genes simultaneously [224]. This system also offers the possibility to generate reporter and conditional alleles in one step [241], and hence speeds up considerably the generation of genetic models in mice. This gene editing method has already been applied to zebrafish [36, 88], and should also make it possible to manipulate the genomes of other mammalian species, including ones that have a frequency range of hearing more similar to that of humans, such as guinea pig or gerbil.

## Abbreviations

Alms1: Alström syndrome 1
aPKC: Atypical protein kinase C
BBS1/4: Bardet–Biedl syndrome 1/4
CEACAM16: Carcinoembryonic antigen-related cell adhesion molecule 16
cKO: Conditional knock-out
CLIC5: Cl^-^ intracellular channel 5
CRISPR/Cas: Clustered regularly interspaced short palindromic repeat/CRISPR-associated
CTHRC1: Collagen triple helix repeat containing 1
cx26: Connexin 26
cx30: Connexin 30
dchs1: Dachsous 1
ELMO: Engulfment and cell motility
ERM: Ezrin/radixin/moesin
eps8: Epidermal growth factor receptor pathway substrate 8
Fat4: FAT tumor suppressor homolog 4
Gα_i_: GTP-binding protein alpha-i subunit
GFP: Green fluorescent protein
GJB2: Gap junction protein beta 2 (connexin 26)
GJB6: Gap junction protein beta 6 (connexin 30)
GPSM2: G-protein signaling modulator 2
ift88: Intraflagellar transport 88 homolog
IHC: Inner hair cell
KCC3/4: K^+^/Cl^-^ cotransporter 3/4
Kcne1: K^+^ voltage-gated channel, Isk-related subfamily, member 1
Kcnj10: K^+^ inwardly rectifying channel, subfamily J, member 10
Kcnq1/4: K^+^ voltage-gated channel, subfamily Q, member 1/4
kif3a: Kinesin family member 3A
KO: Knock-out
LOXHD1: Lipoxygenase homology domains 1
MAGI1: Membrane-associated guanylate kinase inverted 1
MAGUK: Membrane-associated guanylate kinase
MET: Mechano-electrical transduction
MKKS: McKusick–Kaufman syndrome
Mks1: Meckel syndrome, type 1
NHERF1/2: Na^+^/H^+^ exchanger regulatory factor 1/2
NKCC1: Na^+^–K^+^–2Cl^−^ cotransporter
OHC: Outer hair cell
PDZ: Postsynaptic density protein (PSD95), *Drosophila* disc large tumor suppressor (Dlg1) and zonula occludens-1 protein (ZO-1)
PDZD7: PDZ domain containing 7
PMCA2: Plasma membrane Ca^2+^ ATPase 2
PST: Proline-serine-threonine rich domain
PTK7: Protein tyrosine kinase 7
PTPRQ: Protein tyrosine phosphatase receptor Q
rdx: Radixin
ror2: Receptor tyrosine kinase-like orphan receptor 2
scrib: Scribbled
sec24b: Sec24 family member B
smurf1/2: SMAD-specific E3 ubiquitin protein ligase 1/2
TJP2: Tight junction protein 2
TRIOBP: TRIO and F-actin binding protein
TTC8: Tetratricopeptide repeat domain 8
USH: Usher syndrome
vangl1/2: vang-like 1/2
VLGR1: Very large G-coupled receptor 1
ZO-1: Zonula occludens 1
